# The addition of sodium thiosulphate to hyperthermic intraperitoneal chemotherapy with cisplatin in ovarian cancer

**DOI:** 10.1016/j.gore.2021.100796

**Published:** 2021-05-26

**Authors:** Kate Glennon, Karen Mulligan, Kirsten Carpenter, Ruth Mooney, Jurgen Mulsow, Orla McCormack, William Boyd, Tom Walsh, Ruaidhri McVey, Claire Thompson, Brid Ryan, Katie Padfield, Patrick Murray, Donal J Brennan

**Affiliations:** a Department of Gynaecological Oncology, UCD School of Medicine, Mater Misericordiae University Hospital, Dublin 7, Ireland; b Department of Surgery, Mater Misericordiae University Hospital, Dublin 7, Ireland; c National Centre for Peritoneal Malignancy, Mater Misericordiae University Hospital, Dublin 7, Ireland; d Department of Pharmacy, Mater Misericordiae University Hospital, Dublin 7, Ireland; e St. Vincent’s University Hospital, Dublin 4, Ireland; f University College Dublin Clinical Research Centre, Belfield, Dublin 4, Ireland; g Department of Anaesthesia and Peri-operative Medicine, Mater Misericordiae University Hospital, Dublin 7, Ireland; h Systems Biology Ireland, UCD School of Medicine, Belfield, Dublin 4, Ireland

**Keywords:** HIPEC, Epitheal Ovarian Cancer, Cisplatin, Renal toxicity

## Abstract

Cisplatin based hyperthermic intraperitoneal chemotherapy (HIPEC) has been shown to prolong recurrence free and overall survival of women with ovarian cancer who have responded to neoadjuvant chemotherapy. The aim of this study was to assess the impact of cytoreductive surgery with or without the addition of HIPEC on renal function.

**Method:**

This is a retrospective case-controlled study at a tertiary teaching hospital in Dublin, Ireland. All patients who had interval cytoreductive surgery (CRS) and HIPEC from October 2017 to October 2020 were included. A cohort of patients who had interval CRS without HIPEC were included as a control. Sodium thiosulphate (ST) was added to the HIPEC protocol in 2019. In order to assess the impact of ST as a renal protectant, renal function and post-operative outcomes were compared between the groups.

**Results:**

Sixty patients who had interval CRS were included, thirty of whom received cisplatin-based HIPEC. Seven received cisplatin 50 mg/m^2^ without the addition of ST. Twenty three patients received cisplatin 100 mg/m^2^ and ST. There were no statistically differences in age, body mass index BMI, American society of anaesthesia score, estimated blood loss or peritoneal cancer index between the cohorts (p > 0.05). The only episode of acute kidney injury (AKI) was within the HIPEC cohort, after cisplatin 50 mg/m^2^ (without ST) and this was sustained at three months. In contrast, no patients within the CRS cohort or cisplatin 100 mg/m^2^ that received the addition of ST, sustained a renal injury and all had a creatinine within the normal range at three days post operatively.

**Conclusion:**

The renal toxicity associated with cisplatin HIPEC and major abdominal surgery can be minimised with careful preoperative optimisation, intra operative fluid management and attention to renal function. The addition of sodium thiosulphate is a safe and effective method to minimise toxicity and should be added to any cisplatin HIPEC protocol.

## Highlights

• Cisplatin chemotherapy is highly nephrotoxic and is a dose limiting side effect.
• The OVIHIPEC-1 trial employed sodium thiosulphate (ST) as a renal protectant.
• We analyse the implementation of HIPEC for EOC in a peritoneal malignancy centre.
• One acute kidney injury (AKI) event was noted when ST was not used with HIPEC.
• No AKI was observed when sodium thiosulphate was used with cisplatin at 100 mg/m^2.^

## 1 Introduction

Chemotherapy combined with cytoreductive surgery has been the mainstay of approach to the treatment of epithelial ovarian cancer (EOC). The route of chemotherapy administration in EOC has been the subject of much debate. While intraperitoneal chemotherapy (IP) enhances local drug delivery to the peritoneal surface, it has not been widely adopted due to concerns regarding toxicity issues associated with the use of the intraperitoneal catheter (Jaaback and Johnson, 2006). Although historic data demonstrated that adjuvant intraperitoneal chemotherapy was associated with a significant survival benefit (Jaaback and Johnson, 2006, Tewari et al., 2015), recent randomised trials have failed to replicate these findings (Walker et al., 2019). Attention, therefore, has shifted to the use of hyperthermic intra-operative intraperitoneal chemotherapy (HIPEC) in EOC. In addition to CRS, HIPEC has been the cornerstone of treatment in pseudomyxoma peritonei for the last decade (Sugarbaker et al., 1999, chua et al., 1999).

The open-label OVIHIPEC-1 phase 3 randomised control trial provides the most robust evidence to support the use of HIPEC in EOC after complete or near complete (residual disease <2.5 mm) interval cytoreductive surgery (van Driel et al., 2018). Using cisplatin at a dose of 100 mg m^2^ for 90 min at 40 °C, the addition of HIPEC significantly increased recurrence free and overall survival and this has now been incorporated into National Comprehensive Cancer Network (NCCN) guidelines (Armstrong et al., 2019). Adoption of HIPEC has been slow due to concerns regarding morbidity with a particular emphasis on nephrotoxicity which can be a serious and dose limiting side effect of cisplatin.

The mechanism of cisplatin nephrotoxicity is multifactorial, and may be related to pre-existing conditions, blood loss, hydration status and intra-operative fluid management. Cisplatin can also cause direct nephrotoxicity as it accumulates within the renal epithelial cells leading to DNA damage and release of damage associated molecular patterns (DAMPs). As a consequence, cytokines and inflammatory cells are recruited to the kidney. The resultant accumulation of mast cells, neutrophils, macrophages, natural killer (NK) cells and T lymphocytes into the injured kidneys further exacerbates renal damage (Blachley and Hill, 1981). The mechanism of ST mediated nephroprotection is poorly understood, however, it may work as a chelating agent as it can binds to, and chemically inactivates platinum (Elferink et al., 1986) leading to a reduction in renal excretion, and, as a consequence, limits renal tubular cell necrosis (Ceresoli et al., 2016). In addition, ST may also protect against renal magnesium wasting (Coccolini et al., 2015, markman et al., 1986).

OVIHIPEC-1 trial (van Driel et al., 2018) used sodium thiosulphate (ST) as a renal protectant, and as a result, there was only one grade three renal injury in the HIPEC group. This is substantially lower than previous reports suggesting significantly higher rates of renal injury following cisplatin-based HIPEC in the absence of sodium thiosulphate nephroprotection (Gonzalez Bayon et al., 2013, Sin et al., 2017, Sun et al., 2016). The reported incidence of renal injury after HIPEC using cisplatin at 100 mg m^2^ without ST ranges from 0 to 8% (Vaira et al., 2014, Roviello et al., 2006, Gori et al., 2005, Teo et al., 2013, Teo et al., 2017 Dec 1, Tan et al., 2017 May, Cascales-Campos et al., 2014 Aug, Gouy et al., 2016, Perazella, 2012, Sun et al., 2016). Lower doses of cisplatin between 50 and 80 mg/m^2^, without the addition of ST, have also resulted in reports of severe kidney injury in patients who subsequently required dialysis following severe renal injury (Zivanovic et al., 2015, Eckardt and Kasiske, 2009, Kellum and Lameire, 2013, Elferink et al., 1986, Nagai et al., 1995). Side effects including anaphylaxis, metabolic acidosis and prolonged QT interval have been reported but are rare (Laplace et al., 2020).

There is still paucity of evidence to support the use of sodium thiosulphate in HIPEC outside of the OVIHIPEC-1 trial. A number of historical cohort studies have demonstrated that ST may protect against nephrotoxicity following cisplatin-based HIPEC at a dose of at least 100 mg/m^2^ (Ceresoli et al., 2016 Jun, Bakrin et al., 2014, Markman et al., 1985, Ceelen et al., 2012 Jul, Jones et al., 2006, Ghirardi et al., 2020 Dec 15). Zanon et al. used cisplatin at both 100 and 150 mg/m^2^ for 60 min and renal injury was noted in two patients who did not receive ST (Zanon et al., 2004). Ghirardi et al. recently reported on the real-life experience of the implementation of HIPEC using the OVIHIPEC-1 protocol, however did not focus on renal morbidity (Ghirardi et al., 2020). Based on this background, the purpose of this study was to analyse the implementation of HIPEC for EOC within a tertiary peritoneal malignancy centre and the impact of the addition of sodium thiosulphate infusion on renal toxicity.

## 2 Methods

This was a case-controlled study at a tertiary level hospital in Dublin Ireland with significant experience in ovarian cancer cytoreductive surgery and HIPEC. From October 2017 to October 2020, prospective data was collected on consecutive patients with EOC who attended for interval CRS with or without the addition of HIPEC. This review was approved by the Audit Department of the Mater Misericordiae University Hospital.

Following MDT discussion, patients were deemed suitable for interval CRS with or without HIPEC. Inclusion criteria for HIPEC were stage III/IVa EOC, with an ECOG status <2, who had responded to neoadjuvant chemotherapy in whom complete or near complete cytoreduction (residual disease <2.5 mm) was possible. Exclusion criteria included those in whom complete cytoreduction was not feasible, baseline renal dysfunction (creatinine >140 µmmol) or a previous malignancy in the prior 5 years. In order to assess the impact of HIPEC on renal toxicity we compared two cohorts – initially those who had interval CRS without HIPEC with those who received HIPEC with cisplatin. These were age, BMI, ASA and stage matched to the HIPEC cohort. Excluded from this study were patients who had primary CRS and prior treatment with bevacizumab.

All women attended a perioperative preassessment clinic with an anaesthetist. At this, a baseline medical assessment included history and airway assessment, medication review and baseline renal function was performed. All women undergoing interval CRS + HIPEC with cisplatin 100 mg/m^2^ had a standardised bowel preparation with immunonutrition protocol. Those women who had CRS without the addition of chemotherapy received a phosphate enema pre operatively. All patients undergoing CRS with our without HIPEC were pre-emptively admitted to the high dependence unit (HDU) following surgery. Prior to publication of the OVIHIPEC trial in 2018, HIPEC was administered using a dose of 50 mg/m^2^ cisplatin for 60 min (n = 7) without the addition of sodium thiosulphate (see below). The OVIHIPEC trial protocol was adopted in our unit in March 2019 and subsequently all women deemed eligible for HIPEC women received Cisplatin 100 mg/m^2^ over 90 mins with the addition of sodium thiosulphate (n = 23). HIPEC was administered using an open coliseum technique using the SUNCHIP2 system (Gamida, France).

### 2.1 Intra-operative fluid management

Both cohorts of patients were monitored intra operatively with invasive arterial blood pressure (BP), fluid responsiveness index (aiming for pulse pressure variation <12%) and urine output (targeting >1 ml/kg/hr). In order to maintain mean arterial pressure (MAP), norepinephrine infusion was administered in addition to fluid therapy in response to an expected drop in SVR from haemodynamic responses to the procedure and epidural infusion. Colloid or blood were administered if clinically indicated.

### 2.2 Sodium thiosulphate protocol

The protocol was adopted from the OVIHIPEC trial (van Driel et al., 2018) and involved the addition of sodium thiosulfate: 9 g/m^2^ in 200 ml distilled water, made isotonic with sodium chloride 0.9% given IV over 15–20 min, concurrently at start of hyperthermic infusion of cisplatin. The initial bolus was followed by 12 g/m^2.^ thiosulphate IV continuous infusion over 6 h. This was made with 1000 ml of sodium chloride 0.9% and infused at 167 ml/hr. Urine production was closely monitored with an aim that intra-operative urine output should be ≥1 ml/kg/hr throughout the procedure and for 24 h in HDU following surgery. Hourly fluid input and output was documented and 12 hourly renal function was performed as standard.

### 2.3 Data collection and statistical analysis

Baseline laboratory data (baseline serum creatinine, glomerular filtration rate (GFR), and clinical data (histology, stage, age, BMI, peritoneal carcinomatosis index (PCI)) was collected prospectively from the electronic patient record. Intraoperative data (intraoperative solute and fluid management, red cell transfusion) was obtained from the anaesthetic electronic record. Descriptive statistics were generated for clinicopathologic variables, including means, medians, ranges, and standard deviations for continuous data and frequencies and percentages for categorical data respectively. Quantitative variable were assessed with one way ANOVA test across all cohorts and Mann Whitney test was used to compare differences between two groups. Statistical significance was set at *p* < 0.05 for all analysis that were performed using IBM SPSS 24 or Prism Graph Pad.

### 2.4 Classification of renal disease

The definition of AKI was based on the 2012 Kidney disease, improving global outcomes system of renal failure classification (Kellum and Lameire, 2013, Arjona-Sánchez et al., 2016) (Table S1). In addition, absolute increases in serum creatine (sCr) the delta creatinine system was also used: Stage 0, sCr increase <0.3 mg/dL (<26.5 μmol/l), Stage 1, sCr increase 0.3–0.69 mg/dL, Stage 2, sCr increase 0.7–1.19 mg/dL and Stage 3, sCr increase ≥1.2 mg/dL or initiation of renal replacement therapy. Baseline sCr was defined as creatinine at pre-operative assessment or on day of admission. The peak sCr was defined as the highest sCr value reached during hospitalisation. The use of a standardised criteria enables early detection and treatment of patients with AKI.

## 3 Results

A total of sixty women, who attended for interval CRS surgery for advanced ovarian cancer between October 2017 and October 2020, were included in this study. All patients included had three cycles of NACT with carboplatin and paclitaxel. Thirty patients received cisplatin-based HIPEC. A similar age and BMI matched cohort of 30 patients who had interval CRS without HIPEC between Jan 2019 and June 2020 were included as control cases. The clinical characteristics and demographics were summarized in Table 1. In summary, there were no statistically differences in age, BMI, ASA score, estimated blood loss or peritoneal cancer index (PCI) between all cohorts (p > 0.05). The median length of stay for the cytoreductive group was 7 days (IQR 3–9.5) and 13 days (IQR 8.5–19) for HIPEC patients. Intraoperative fluid management, PCI and EBL also remained stable across both HIPEC cohorts (Table 1). Among the patients who received cisplatin, twenty three had HIPEC with 100 mg/m^2^ cisplatin for 90 min with ST and seven patients had HIPEC with 50 mg/m^2^ cisplatin for 60 min without ST.

**Table 1:** Demographic and intraoperative Data: All cohorts.

	CRS with HIPEC n = 30	CRS No chemotherapy n = 30	P value chi2
Median Age (IQR) years	55 (46–62)	55.5 (38–85)	0.21
Median BMI (IQR) kg/m2	24 (19–28)	25.8 (21–37)	0.22
Number with BMI > 25	10 (33%)	12 (40%)	
ASA score (Median ± SD)	2 (±0.49)	2 (±0.50)	0.7
ECOG status -median (range)	0 (0–1)	0 (0–2)	
PCI (median IQR)	15 (7–12)	12 (3–14)	0.4
FIGO stage- median (range)	IIIC (IIIC(- IVB)	IIIC (IIB - IVB)	

**Intraoperative Fluid Management** Median (+IQR)			
Hartmans ml	1000 (1000–3400)	3400 (900–5000)	0.3
Gelofusion ml	950 (0–1050)	750 (500–1375)	0.26
Intraoperative Red Cell Concentrate (units)	1 (0–1.75)	2 (1–2)	0.6
Total operative urine output ml	937.5 (210–1154.5)	375 (181–1091)	0.22
Estimated intraoperative blood loss ml	3500 (1000–1778)	1150 (900–1750)	0.24

CoMorbidities	CRS with HIPEC n = 30	CRS No chemotherapy n = 30
Diabetes	5 (7%)	0
Renal Failure	1 (3.3%)	1 (3.3%)
Cardiac	1 (3.3%)	1 (3.3%)
Respiratory	5 (16.6%)	3 (10%)

Hypoalbuminaemia was observed in all cohorts on day three post operatively (Table 2). No acute acid-base disturbances were identified in the first 24 post-operative hours. No allergic reaction to sodium thiosulphate was observed in this study. Electrolyte disturbances in the form of hypocalcemia, hypokalemia, hypophosphatemia, and hypomagnesemia are commonly seen in recovery phase of renal injury (Blachley and Hill, 1981, Lajer et al., 2003). Three patients developed hypomagnesemia (<0.70 mmol/l) within the first 24 h of HIPEC (3/30, 10%) with one of these in the cisplatin 50 mg/m^2,^ cohort who did not receive ST. Serum creatinine and GFR remained within normal limits in all of these patients up to post-operative day three.

**Table 2:** Laboratory data of CRS with and without HIPEC.

Cytoreduction and Chemotherapy with Cisplatin			Cytoreduction with no chemotherapy	
Median	Cisplatin 50 mg/m^2^ n = 7	Cisplatin 100 mg/m^2^ n = 23	CRS no chemo n = 30	P value (ANOVA)
Baseline creatinine	69 (17.4)	63.5 (11.7)	66 (11.9)	0.09
Day 1 creatinine	66 (23.9)	60.5 (12.9)	63 (13.2)	0.14
Day 3 creatinine	55 (20.4)	51.5 (12.3)	57 (13.8)	0.10
3 month follow up	55* (27.6)	69* (13.6)	58* ( 12.3)	0.98
Baseline urea	4 (1.2)	4.35 (1.4)	4.15 (0.7)	0.65
Day 1 urea	4.3 (0.5)	3.9 (0.9)	3 (1.0)	0.015
Day 3 urea	4.8 (2.1)	4.05 (1.66)	3.45 (1.4)	0.15
Baseline Albumin	38 (8.9)	34 (7.62)	41.5 (7.18)	0.32
Day 3 Albumin	21 (3.2)	28 (5.31)	26.5 (5.5)	0.08
pH at 12hrs	7.40 (0.05)	7.33 (0.04)	7.4 (0/05)	0.28
pH at 24hrs	7.38 (0.03)	7.34 (0.05)	7.4 (0.2)	0.0004

Lactate at 12 hrs	2.1 (1.25)	1.9 (1.08)	1.2 (0.6)	0.08
Lactate at 24 hrs	2.2 (1.34)	1.4 (0.84)	1.3 (0.5)	0.10

Median ± SD.

Creatinine = μmmol/l.

Urea – mmol/l.

Albumin = g/l.

*Data available for: • Cisplatin 50–5/7 patients • Cisplatin 100–6/23 • CRS no chemo – 12/30.

**Normal ranges**

Creatinine (46–86 μmmol).

Urea (2.8–8.6).

Albumin (35–50).

Ph (7.35–7.45).

Lactate (0.5–2).

### 3.1 Acute renal failure

Within the thirty patients who had CRS, no patient had an AKI as per the KDIGO definition. One patient had a grade 3a chronic renal failure prior to CRS. Six (20%) experienced a rise in creatinine on day one (median rise in creatinine = 10 µmmol, SD = ±3.2) with two of these having a creatinine above the normal range (>86 µmmol). At day three, only the patient with pre-existing chronic renal failure had a persistently high serum creatinine

Within the HIPEC group, one patient developed AKI. This patient had a normal baseline renal function and no co morbidities (ECOG status was 0). She received cisplatin 50 mg/m^2^ for 60 min without ST. Her magnesium was high when tested on day one (0.83 mmol/l) and had normalised when retested day ten. She had a persistently high creatinine at day three (133 µmmol) and again at three months (108 µmmol) and subsequently developed stage 3 CKD. She remains disease free on follow up (36 months). Four (4/7, 57.1%) patients who received 50 mg/m^2^ cisplatin did have a rise in creatinine within a 48 h period (median rise in creatinine = 7 µmmol, SD ± 22.7)). This did not reach the KDIGO criteria for AKI (Increase in SCr ≥ 26.4 µmol/L within 48 h). By day three, all creatinine levels decreased, however two patients (28.5%) having a persistently high creatinine (>86 µmmol) above the normal limit.

**Fig. 1:**
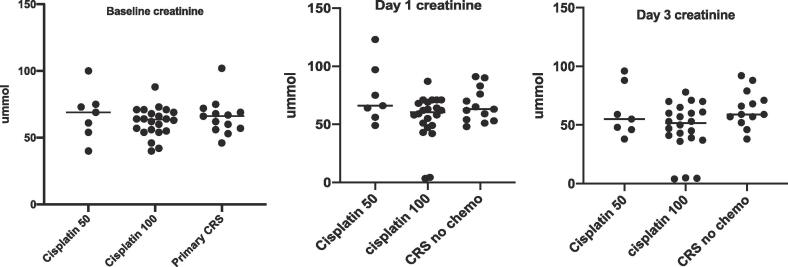
Median Creatinine Results: Baseline, Day 1, Day 3.

No patients within the cisplatin 100 mg/m^2^ with ST cohort developed AKI. (Eleven (47.8%) experienced a small rise in creatinine levels within a 48 h period (median rise = 6 µmmol ± SD 3.53) and the remainder experienced a fall or stable creatinine (median fall = 9 µmmol ± SD 7.9). By day three, only three patients had a persistent rise in creatinine from baseline (3/23, 13%) however this median rise of 3µmmol, did not classify any of these patients as reaching criteria diagnostic for sustained AKI. No patients had a creatinine level outside the normal range (>86 µmmol) at 48 h (Fig. 1).

### 3.2 Delta creatinine

There was one AKI as defined by the delta creatinine system with the peak sCr occurring at day 7. This was in a patient who did not receive ST with 50 mg cisplatin. The median peak rise in creatinine was 9 µmmol within the HIPEC group equating to stage 0 delta creatinine. In the cisplatin 50 group, the delta creatine was negative 21 µmmol in three patients (3/7, 42.8%) and a negative 12 µmmol in 18 of the cisplatin 100 cohort.

### 3.3 MAKE 30 (Major adverse kidney event at 30 days)

Persistent renal dysfunction was seen at 30 days, in two patients, one of whom received cisplatin 50 mg/m^2^, administered without the addition of ST, the other had CRS surgery and grade 3a CKD prior to surgery. Data on renal function was available in 25 patients, 8 patients in the CRS group, and 17 in the HIPEC group. Within the CRS cohort, there was a small rise in serum creatinine in 4 of these from day one levels (median rise = 12 µmmol). Within the cisplatin cohort, 5 had a small rise in creatine ( median = 5 µmmol) also not reaching criteria for chronic kidney disease. There was no mortality within a 30 day period in either cohort.

### 3.4 Chronic renal impairment

Renal laboratory data was available for 11 (11/30, 36.6%) patients in the HIPEC group and twelve patients (12/30, 40%) in the CRS group at three month follow up. One case of new onset chronic kidney disease (eGFR loss > 25%) at 3 months was observed as described above. Two patients had abnormally low GFR (GFR 45–59) when tested pre operatively. However, both had normal GFR and renal function within the three month period prior to surgery and both patients renal function improved and had normalised by four weeks post operatively. One of these patients developed progressive EOC disease and died on day 79 post operatively.

## 4 Discussion

Herein we explore the incidence of AKI and CKD in a cohort of patients undergoing CRS with and without HIPEC. Importantly, following adaptation of the OVIHIPEC trial protocol, which resulted in double the dose of cisplatin from 50 to 100 mg/m^2^ and the addition of ST as a renal protectant, we describe no evidence of AKI or CKD.

Despite the proven survival benefit with HIPEC (van Driel et al., 2018), multiple high profile contributors have suggested that this is a highly morbid procedure (Vergote et al., 2019). However it is often overlooked that CRS with or without the addition of HIPEC is a complex surgical procedure requiring careful patient selection and meticulous perioperative care. Validation of clinical trial data using real life data and experience of any new protocol is important given the inherent selection bias of all randomised control trials. Therefore although the numbers included in this study are relatively small, they provide important evidence of the OVIHIPEC protocol is not associated with increased renal morbidity outside of a trial protocol. This is important as cisplatin included nephrotoxicity is multifactorial and factors such as age, BMI and use of other nephrotoxic agents may be important contributors to renal injury which may not be reflected in clinical trials with strict inclusion criteria. (Arjona-Sánchez et al., 2016).

As a consequence, all patients in our unit are pre operatively assessed with close attention to baseline renal function medications and ECOG status. As per the OVIHIPEC-1 protocol for use of ST, careful dose adjustment of 33% was made in those patients in whom nephrotoxicity with cisplatin is high risk (age > 65, BMI > 40). During the HIPEC procedure, large volume changes occur with the removal of ascites, resection of the peritoneum and fluid loss from a laparotomy incision. In addition, peritoneal inflammation and heat-induced fluid losses during administration of chemotherapeutic agents at 41C cause electrolyte and cytokine changes and a hypermetabolic state. This induces a state similar to sepsis with a fall in systemic vascular resistance (SVR) and a compensatory increase in heart rate (HR) and cardiac output (CO) placing the patient at risk of tissue hypoperfusion (Shime et al., 1994).

In HIPEC cases, a more liberal fluid strategy of 10–15 ml/kg/hr has often been used in comparison to 6–8 ml/kg/hr for other major abdominal surgery and 4 ml/kg/hr for restricted or goal-directed fluid therapy (Bezu et al., 2020). The role of fluid administration strategies and their impact on AKI is not yet clearly defined in the literature. Two studies that examined the relationship between fluid volume and AKI found no correlation, rather suggesting that any AKI is more likely to be due to the nephrotoxic agents themselves (Shiralkar et al., 2017, Owusu-Agyemang et al., 2012). A recent systematic review, however, does suggest goal-directed fluid therapy with a more restrictive approach, to minimise complications related to fluid overload, is associated with less postoperative morbidity and mortality (Bezu et al., 2020). Our results show that our fluid administration volumes fall within the range of 8–10 ml/kg/hr, in line with experience at other institutions.

This study has a number of limitations. The patient cohort is small. Certain clinical laboratory variables and a three month follow up of renal function was not standardised across all patients. However, only one patient developed severe AKI with HIPEC and this was prior to the addition of sodium thiosulphate using a lower dose of cisplatin than that currently employed in many units since the publication of the OVIHIPEC-1 trial and provides further evidence to support this protocol as the OVIHIPEC-2 trial begins to recruit (Koole et al., 2020). This aims to determine whether primary CRS with HIPEC in EOC improves outcomes compared to primary CRS alone.

A combined approach, incorporating careful intra operative fluid management and attention to renal function, alongside the use of sodium thiosulphate infusion during and after HIPEC (100 mg/m^2^ of cisplatin for 90 min), can help minimise the severe consequences associated with renal damage.

## Declaration of Competing Interest

The authors declare that they have no known competing financial interests or personal relationships that could have appeared to influence the work reported in this paper.
