# Supplementary material for: The addition of sodium thiosulphate to hyperthermic intraperitoneal chemotherapy with cisplatin in ovarian cancer
Source: Gynecol Oncol Rep. 2021 May 26;37:100796. doi: 10.1016/j.gore.2021.100796 (PMC8185237; doi:10.1016/j.gore.2021.100796)
Supplement: Supplementary Table 1 [file mmc1.docx]

Supplementary Table

Table S1

KDIGO definition of Acute Kidney Injury

Adapted from : 2012 Acute Kidney Injury Guideline KDIGO Clinical Practice Guideline for Acute Kidney Injury

Vol 2: 1: March 2012

| **KDIGO Acute Kidney Injury** | **Increase in SCr≥26.4µmol/L within 48h**  **Or ≥50% within 7 d** |
| --- | --- |

| Stage 1 | Increase in SCr≥26.4µmol/L in 48 h (>0.3 mg/dl )  Or increase ≥1.5 x baseline | <0.5ml/kg/h  for ≥6h |
| --- | --- | --- |
| Stage 2 | Increase in SCr ≥2 x baseline | <0.5ml/kg/h for≥12 h |
| Stage 3 | Increase in SCr ≥3 x baseline  Or increase in SCr to ≥354µmol/L  Or initiation of RRT irrespective of SCr  Or in patients<18 years, decrease in eGFR to<35 ml/min per 1.73 m^2^ | <0.3ml/kg/h≥24h or anuria≥12 |
